# 

**Published:** 1874-06

**Authors:** 


### Books and Pamphlets Received.

Materia Medica for the Use of Students. By John B. Biddle, M. D., etc. Sixth Edition Revised and Enlarged with Illustrations. Philadelphia: Lindsay and Blakiston, 1874. Buffalo: Theo Butler & Son.

Hints on the Obstetric Procedure. The Annual Address before the Philadelphia County Medical Society. By William B. Atkinson, M. D., Retiring Pres dent. Delivered May 8th, 1874.

Archives of Electrology and Neurology, a Journal of Electro Therapeutics and Nervous Diseases. Edited by Geo. M. Beard, A. M., M. D.

PUBLISHED MONTHLY,

Containing Original Articles, Reports of Medical Societies and Hospitals, Editorial, Reviews, Correspondence, Army News, etc., etc ; including the usual variety of Medical Periodical Publications. Specimen copies sent on application.

Address Buffalo Medical & Surgcal Journal, Buffalo, N. Y.

TERMS OF SUBSCRIPTION, - - $3.00 PER YEAR, IN ADVANCE.

